# Supplementary material for: The Cholesterol-Modulating Effect of the New Herbal Medicinal Recipe from Yellow Vine (Coscinium fenestratum (Goetgh.)), Ginger (Zingiber officinale Roscoe.), and Safflower (Carthamus tinctorius L.) on Suppressing PCSK9 Expression to Upregulate LDLR Expression in HepG2 Cells
Source: Plants (Basel). 2022 Jul 13;11(14):1835. doi: 10.3390/plants11141835 (PMC9318486; doi:10.3390/plants11141835)
Supplement: Supplementary file 1 [file plants-11-01835-s001.zip › plants-1802126-supplementary.pdf]

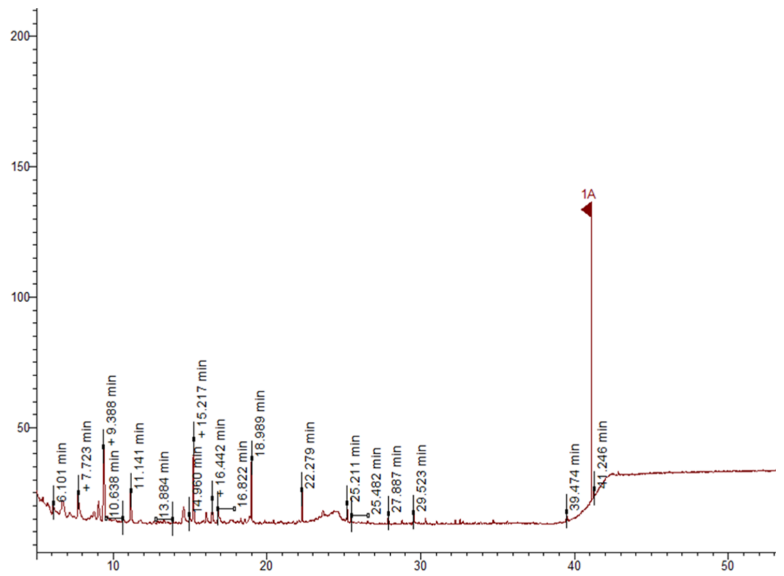

Figure S1 GC-MS/MS chromatogram of water extract of flower of safflower (*Carthamus tinctorius* L.) with the corresponding mass spectrum for peak at retention time of safflower discussed in this study are: 4H-Pyran-4-one, 2,3-dihydro-3,5-dihydroxy-6-methyl- (7.72 min), Benzofuran, 2,3-dihydro- (9.38 min), Cyclohexasiloxane, dodecamethyl- (11.14 min), 3-Isopropoxy-1,1,1,7,7,7-hexamethyl-3,5,5-tris(trimethylsiloxy)tetrasiloxane (15.22 min), 3,4-Dihydroxyphenylglycol, 4TMS derivative (18.99 min).

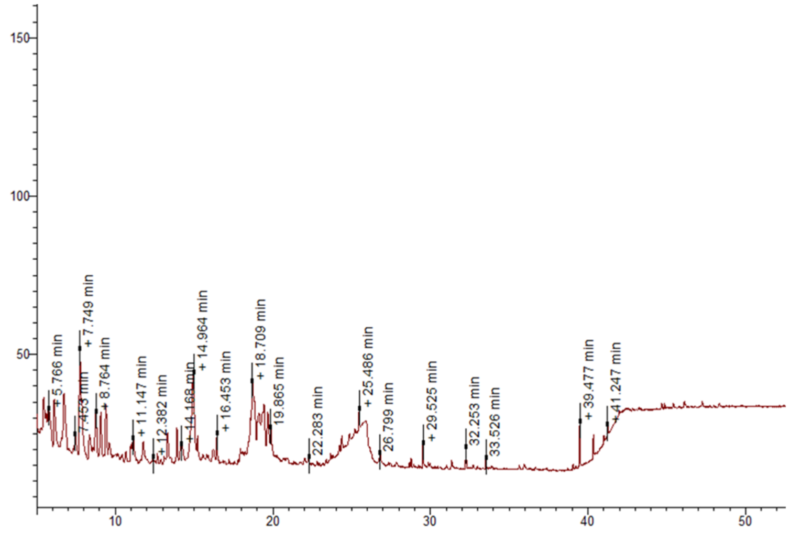

Figure S2 GC-MS/MS chromatogram of ethanolic extract of flower of safflower (*Carthamus tinctorius* L.) with the corresponding mass spectrum for peak at retention time of safflower discussed in this research are: 3-Deoxy-d-mannonic acid (19.44 min), 4H-Pyran-4-one, 2,3-

dihydro-3,5-dihydroxy-6-methyl- (7.75 min), Cyclopentanol (6.74 min), Guanosine (14.97 min), l-Pyrrolid-2-one, N-carboxyhydrazide (14.89 min).

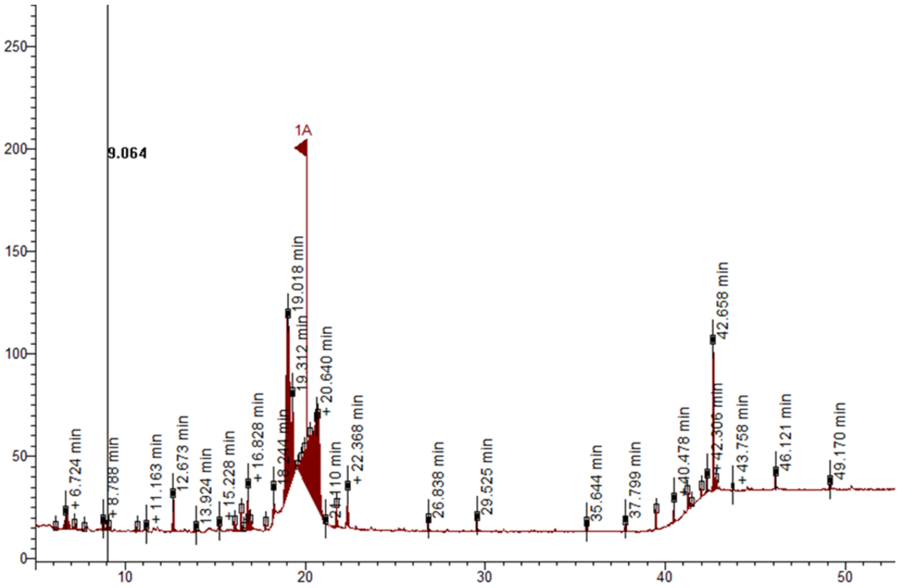

Figure S3. GC-MS/MS chromatogram of water extract of heartwood of yellow vine (*Coscinium fenestratum* (Goetgh.) with the corresponding mass spectrum for peak at retention time of safflower discussed in this research are: Tetraacetyl-d-xylonic nitrile (19.02 min), Inositol, 1-deoxy- (20.64 min), d-Gala-l-ido-octonic amide (19.63 min), Thieno[2,3-b]pyridine,3-amino-2-(3,3-dimethyl-3,4-dihydroisoquinolin-1-yl)-4,6-dimethyl- (42.66 min), Megastigmatrienone (17.81 min).

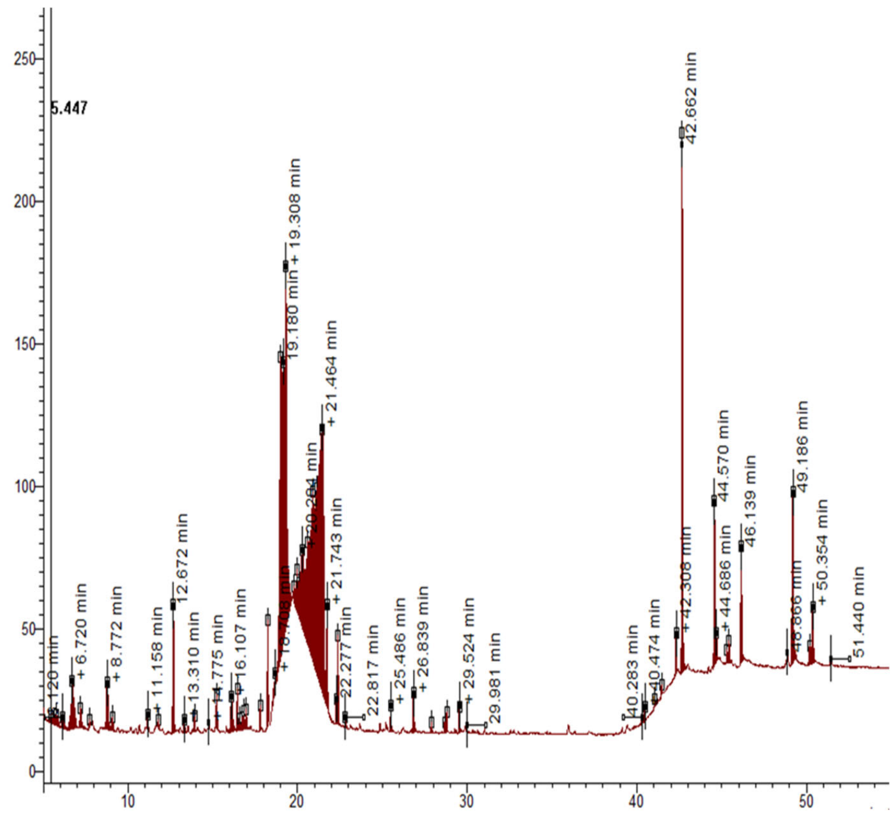

Figure S4. GC-MS/MS chromatogram of ethanolic extract of heartwood of yellow vine (*Coscinium fenestratum* (Goetgh.) with the corresponding mass spectrum for peak at retention time of safflower discussed in this research are: Inositol, 1-deoxy (21.13 min), Megastigmatrienone (19.32 min), Tetraacetyl-d-xylonic nitrile (19.18 min), (E)-2,6-Dimethoxy-4-(prop-1-en-1-yl)phenol (20.90 min), d-Gala-l-ido-octonic amide (20.28 min).

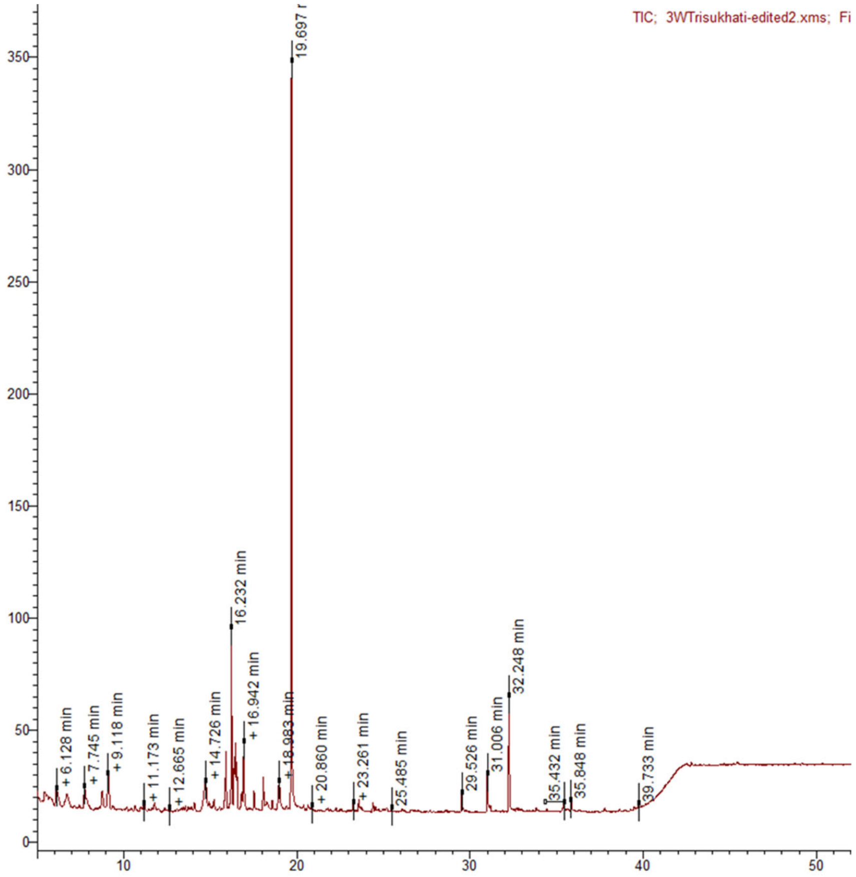

Figure S5. GC-MS/MS chromatogram of water extract of rhizome of ginger (*Zingiber officinale* Roscoe.) with the corresponding mass spectrum for peak at retention time of safflower discussed in this research are: 2-Butanone, 4-(4-hydroxy-3-methoxyphenyl)- (19.70 min), (1S,5S)-2-Methyl-5-((R)-6-methylhept-5-en-2-yl)bicyclo[3.1.0]hex-2-ene (16.24 min), 1-(4-Hydroxy-3-methoxyphenyl)dec-4-en-3-one (32.25 min), 2-Formyl-9-[.beta.-d-ribofuranosyl]hypoxanthine (14.72 min), (1S,5S)-4-Methylene-1-((R)-6-methylhept-5-en-2-yl)bicyclo[3.1.0]hexane (16.95 min).

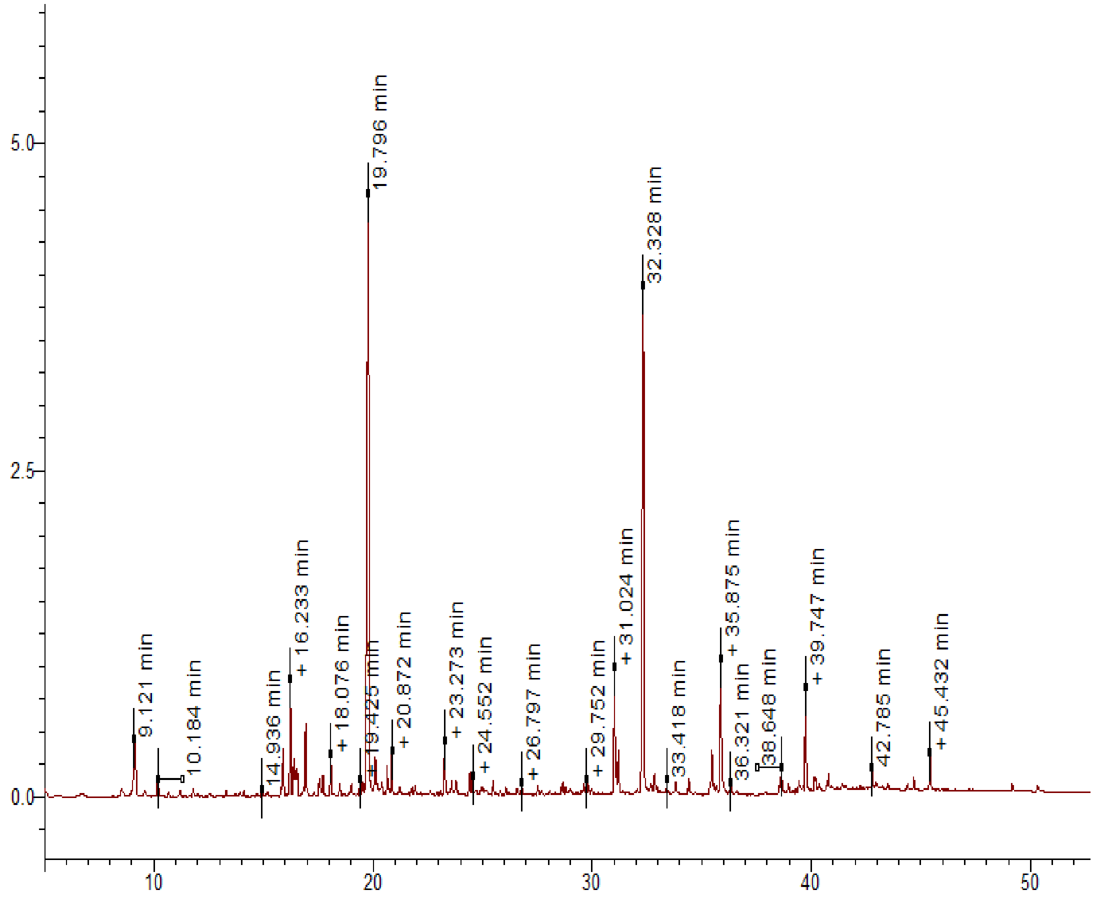

Figure S6. GC-MS/MS chromatogram of ethanolic extract of rhizome of ginger (*Zingiber officinale* Roscoe.) with the corresponding mass spectrum for peak at retention time of safflower discussed in this research are: Butan-2-one, 4-(3-hydroxy-2-methoxyphenyl)- (19.80 min), 1-(4-Hydroxy-3-methoxyphenyl)dec-4-en-3-one (32.33 min), 1-(4-Hydroxy-3-methoxyphenyl)dodec-4-en-3-one (35.87 min), (E)-1-(4-Hydroxy-3-methoxyphenyl)dec-3-en-5-one (31.02 min), 1-(4-Hydroxy-3-methoxyphenyl)tetradec-4-en-3-one (39.74 min).
